# Efficient gold(I)/silver(I)-cocatalyzed cascade intermolecular N-Michael addition/intramolecular hydroalkylation of unactivated alkenes with α-ketones

**DOI:** 10.3762/bjoc.7.126

**Published:** 2011-08-11

**Authors:** Ya-Ping Xiao, Xin-Yuan Liu, Chi-Ming Che

**Affiliations:** 1 Shanghai-Hong Kong Joint Laboratory in Chemical Synthesis, Shanghai Institute of Organic Chemistry, The Chinese Academy of Sciences, 345 Lingling Road, Shanghai 200032 , P. R. China; 2 Department of Chemistry, State Key Laboratory of Synthetic Chemistry, and Open Laboratory of Chemical Biology of the Institute of Molecular Technology for Drug Discovery and Synthesis, The University of Hong Kong, Pokfulam Road, Hong Kong, P. R. China

**Keywords:** cascade, cocatalyzed, gold(I)-catalyzed, intramolecular hydroalkylation, intermolecular N-Michael addition, pyrrolidine, silver(I)-catalyzed

## Abstract

The gold(I)/silver(I)-cocatalyzed cascade intermolecular N-Michael addition/intramolecular hydroalkylation reaction offers a simple and efficient method for the synthesis of pyrrolidine derivatives in moderate to excellent product yields and with moderate to good diastereoselectivities. The reaction conditions and the substrate scope of this reaction are examined, and a possible mechanism involving AgClO_4_ catalyzed intermolecular N-Michael addition and the subsequent gold(I)-catalyzed hydroalkylation is proposed.

## Introduction

Gold complexes are presently receiving a surge of interest in the field of metal-catalyzed organic reactions. They have been shown to be versatile and efficient catalysts for the promotion of a large number of organic transformations, most of which are based on the propensity of gold ion to act as a soft and carbophilic Lewis acid to activate unsaturated C–C bonds towards nucleophilic attack [1–10] (for selected reviews on gold-catalyzed reactions see [1–9]). Based on this mode of activation, several methods for the gold-catalyzed inter- and intramolecular addition of oxygen- [11–15], nitrogen- [10,16–18] (for recent reviews on gold-catalyzed hydroamination see [16–18]), or carbon-nucleophiles [19–24] to unactivated alkenes [21–25] have been developed. On the other hand, in recent years, considerable efforts have been devoted to the development of dual-metal-catalyzed reactions as new strategies for the synthesis of organic compounds with intriguing diversity and selectivity [26–29] (for reviews on cooperative catalysis see [26–27] and for a general review on cocatalysis see [28]). This type of reaction could have the advantages of the combined characteristic features of two metals, often displaying unique reactivity, have a shortened synthetic route and generate less chemical waste. All of these features are of significant economic and environmental benefit. In this context, extensive studies have been conducted on the design and utilization of dual-metal catalyst systems in organic synthesis [26–40] (for recent examples of Au/Pd-cocatalysis see [30–33]; for Au/Mo-cocatalysis see [31,35]; for Au/Ag-cocatalysis see [36–38]; for Au/Yb-cocatalysis see [39] and for Au/Rh-cocatalysis see [40]). However, the use of homogeneous gold catalysts in cooperation with other metal catalysts has been reported only in a few cases [30–40]. In this work, we describe a highly efficient gold(I)/silver(I)-cocatalyzed cascade intermolecular N-Michael addition/intramolecular hydroalkylation process. A variety of pyrrolidine compounds were conveniently prepared in moderate to excellent yields and with moderate to good diastereoselectivities from the simple starting materials.

**Scheme 1:**
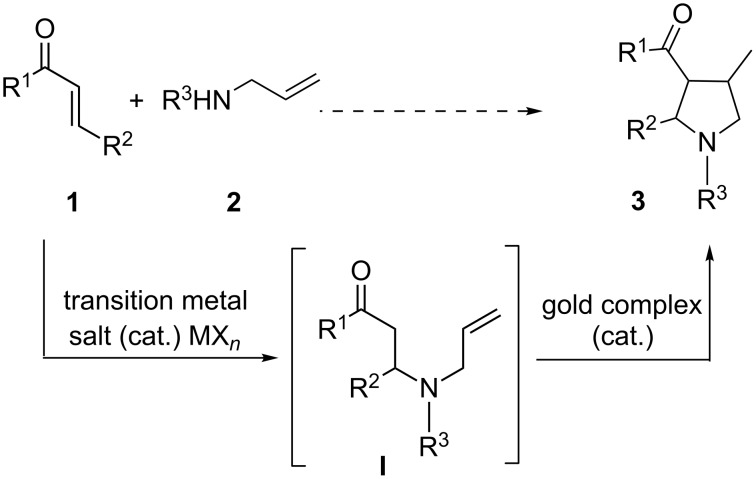
Cascade intermolecular N-Michael addition/intramolecular hydroalkylation of unactivated alkenes with α-ketones catalyzed by a dual-metal catalytic system comprising a transition metal salt and a gold(I) complex.

More recently, we have reported that gold(I) complexes can efficiently catalyze direct intramolecular hydroalkylation of unactivated alkenes with α-ketones, via the exo-trig cyclization, to build a variety of new five- and six-membered rings [24]. However, all of the substrates examined in this gold(I)-catalyzed reaction were prepared and isolated prior to use, and this is not desirable as the synthesis of these substrates could be tedious and time-consuming. The increasing demand for environmentally benign and economical synthetic processes calls for the development of cascade reactions for the efficient construction of cyclic compounds from simple starting materials [41]. We initially envisioned that the gold(I)-catalyzed cascade process could be established starting from the intermolecular N-Michael reaction of α,β-unsaturated ketone **1** and substituted allylamine **2** to furnish an α-ketone intermediate **I** [42–44] (for gold-catalyzed intramolecular N-Michael reaction see [42–43]), which further undergoes a subsequent gold(I)-catalyzed hydroalkylation to give pyrrolidine compounds **3** (Scheme 1); these compounds are versatile synthetic building blocks for organic synthesis and are important structural elements of many therapeutic drug molecules. Disappointingly, no conversion was observed when (*t*-Bu)_2_(*o*-diphenyl)PAuOTf only was used as the catalyst. Since dual-metal-catalysis is of interest from the perspective of unique reactivity [26–40], we explored a new cascade reaction involving intermolecular N-Michael addition catalyzed by an appropriate transition metal salt and subsequent intramolecular hydroalkylation catalyzed by a gold complex (Scheme 1).

## Results and Discussion

The optimization of the reaction conditions was performed using the reaction of phenyl vinyl ketone (**1a**) with *N*-tosylallylamine (**2a**) in the presence of 5 mol % of (*t-*Bu)_2_(*o*-diphenyl)PAuOTf. However, no desired product **3a** was observed (Table 1, entry 1). When using a combination of 5 mol % of (*t-*Bu)_2_(*o*-diphenyl)PAuCl and AgOTf as catalyst, the corresponding product **3a** was obtained in 25% yield (Table 1, entry 2) (a small amount of silver salt may have remained in the reaction system when the mol ratio of silver salt to gold complex was 1:1, see [45]). Upon further increase of the AgOTf loading to 10 mol %, the corresponding product **3a** was formed in 58% yield with a diastereomeric ratio of 5.4:1 (Table 1, entry 3) [46]. Using a combination of 5 mol % of (*t*-Bu)_2_(*o*-diphenyl)PAuCl and 15 mol % of AgOTf as a dual-metal catalyst system lead to the formation of pyrrolidine derivative **3a** as a 5.3:1 mixture of two diastereomers in 67% yield (Table 1, entry 4). The yield increased from 58% to 67% as the mol ratio of **2a** to **1a** was increased from 1.2/1 to 1.5/1 (Table 1, entries 4 and 5). However, the yield did not increase remarkably when the mol ratio of **2a** to **1a** was raised from 1.5/1 to 2.5/1 (Table 1, entries 4–7). As depicted in Table 1, varying the method of the addition of phenyl vinyl ketone (**1a**) to the reaction mixture did not have a noticeable effect on the yield of **3a** (Table 1, entries 8–11).

**Table 1:** The optimization of the reaction conditions.

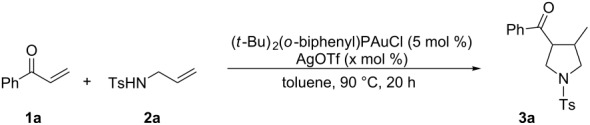				

entry^a^	x (mol %)	mol ratio (**2a**/**1a**)	*trans*/*cis*^b^	yield (%)^b^

1^c^	5	1.5/1	–	–^d^
2	5	1.5/1	1.4:1	25
3	10	1.5/1	5.4:1	58
4	15	1.5/1	5.3:1	67
5	15	1.2/1	5.7:1	58
6	15	2.0/1	5.9:1	66
7	15	2.5/1	5.5:1	64
8	15	1/1.5	5.5:1	64
9^e^	15	1/1.5	5.2:1	60
10^f^	15	1/1.5	5.5:1	65
11^g^	15	1/1.5	5.5:1	64

^a^Reactions were carried out in toluene (0.5 mL) at 0.25 mmol scale based on **1a** or **2a**, **1a** was added in one portion. ^b^Yield and selectivity were determined by ^1^H NMR spectroscopy (internal standard: trimethyl(phenyl)silane). ^c^5 mol % of (*t*-Bu)_2_(*o*-diphenyl)PAuOTf was used as catalyst. ^d^no desired product **3a** was detected. ^e^**1a** (dissolved in 0.3 mL of toluene) was added dropwise over 6 h. ^f^**1a** was added in two portions every 3 h. ^g^**1a** was added in three portions every 2 h.

To identify further the optimal reaction conditions for the gold(I)/silver(I)-cocatalyzed cascade reaction, a number of dual-metal catalyst systems, composed of 15 mol % of silver salt with 5 mol % of gold(I) complex in different organic solvents, were tested in the reaction of phenyl vinyl ketone (**1a**) with 1.5 equiv of *N*-tosylallylamine (**2a**) (Table 2). AgClO_4_ was found to be the best silver salt for this reaction (Table 2, entries 1–5). A panel of Au(I) complexes with different ancillary ligands was also screened for activity and diastereo-induction in this cascade reaction (Table 2, entries 5–9). Among the complexes examined, (*t*-Bu)_2_(*o*-biphenyl)PAuCl gave the best result (Table 2, entry 5). Further screening of solvents revealed that toluene gave the best result, while the other solvents, dioxane, nitromethane, 1,2-dichloroethane, tetrahydrofuran, benzene, and acetonitrile, gave low product yields and low diastereoselectivity (Table 2, entries 5 and 10–15). After optimization of the reaction conditions, the protocol with the combination of 5 mol % of (*t*-Bu)_2_(*o*-biphenyl)PAuCl and 15 mol % of AgClO_4_ as a dual-metal catalyst system at 90 °C in toluene for 20 h gave the product **3a** in 76% yield.

**Table 2:** Screening catalysts and solvents.

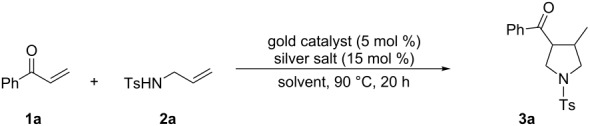				

entry^a^	gold catalyst/silver salt	solvent	*trans*/*cis*^b^	yield (%)^b^

1	(*t*-Bu)_2_(*o*-biphenyl)PAuCl/AgOTf	toluene	5.3:1	67
2	(*t*-Bu)_2_(*o*-biphenyl)PAuCl/AgSbF_6_	toluene	2.2:1	35
3	(*t*-Bu)_2_(*o*-biphenyl)PAuCl/AgPF_6_	toluene	2.6:1	62
4	(*t*-Bu)_2_(*o*-biphenyl)PAuCl /AgBF_4_	toluene	–	<5
**5**	**(*t*-Bu)_2_(*o*-biphenyl)PAuCl/AgClO_4_**	**toluene**	**4.1:1**	**76**
6	Ph_3_PAuCl/AgClO_4_	toluene	–	<5
7	Cy_3_PAuCl/AgClO_4_	toluene	–	<5
8	IPrAuCl/AgClO_4_^c^	toluene	4.4:1	63
9	L^1^AuCl/AgClO_4_^d^	toluene	3.1:1	50
10	(*t*-Bu)_2_(*o*-biphenyl)PAuCl/AgClO_4_	dioxane	4.0:1	63
11	(*t*-Bu)_2_(*o*-biphenyl)PAuCl/AgClO_4_	CH_3_NO_2_	2.8:1	47
12^e^	(*t*-Bu)_2_(*o*-biphenyl)PAuCl/AgClO_4_	DCE	1.8:1	34
13^e^	(*t*-Bu)_2_(*o*-biphenyl)PAuCl/AgClO_4_	THF	1.8:1	76
14^e^	(*t*-Bu)_2_(*o*-biphenyl)PAuCl/AgClO_4_	benzene	3.2:1	60
15^e^	(*t*-Bu)_2_(*o*-biphenyl)PAuCl/AgClO_4_	CH_3_CN	–	<5

^a^Reactions were carried out in toluene (0.5 mL) at 0.25 mmol scale, **1a** (0.25 mmol) and **2a** (0.375 mmol) were added in one portion. ^b^Yield and selectivity were determined by ^1^H NMR spectroscopy (internal standard: trimethyl(phenyl)silane). ^c^IPr= *N*,*N*’-bis(2,6-diisopropylphenyl)imidazol-2-ylidene. ^d^L^1^ = (Cy)_2_(2',4',6'-triisopropyl-*o*-biphenyl)P. ^e^Reactions were carried out under reflux.

With the optimal reaction conditions, we next explored the substrate scope with the protocol for the Au(I)/Ag(I)-cocatalytic system (Table 3). For example, treatment of substrate **1b**, which has an electron-donating *para*-methoxy group on the phenyl ring, with **2a** under the optimized reaction conditions gave the expected product **3b** in 92% yield, albeit with no diastereoselectivity (Table 3, entry 2). In addition to substrate **1b**, α,β-unsaturated ketone **1c**, with electron-withdrawing substituent on the phenyl ring, underwent this cascade reaction to afford the corresponding product **3c** in 58% yield with a diastereomeric ratio of 1.7:1 (Table 3, entry 3). Reaction of alkyl α,β-unsaturated ketone **1d** in the presence of 5 mol % of (*t*-Bu)_2_(*o*-biphenyl)PAuCl and 15 mol % of AgClO_4_ also gave the desired product **3d** in 92% yield with a diastereomeric ratio of 5.5:1 (Table 3, entry 4). Other substituted allylamines were also examined. A series of substituted allylamines with 4-nitrobenzenesulfonyl group and 2,4,6-triisopropylbenzenesulfonyl group were similarly treated with alkyl α,β-unsaturated ketone **1d**, and the corresponding products **3e** and **3f** were obtained in moderate to excellent yields with similar diastereomeric ratios of around 5.2:1 (Table 3, entries 4–6). Notably, the gold(I)/silver(I)-cocatalyzed cascade reaction was also successfully applied to furnish spirocyclic pyrrolidine derivative **3g** in 52% yield starting from the readily available precursor 2-methylene-3,4-dihydronaphthalen-1(2*H*)-one (**1e**) and *N*-tosylallylamine (**2a**) (Table 3, entry 7).

**Table 3:** Cascade synthesis of pyrrolidine catalyzed by a dual-metal catalytic system comprising of gold(I) and silver(I) catalysts.

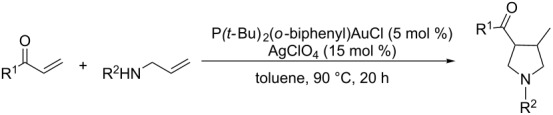					

entry^a^	α,β-unsaturated ketone	substituted allylamine	major product	dr^b^	yield (%)^b^

1	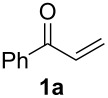	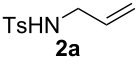	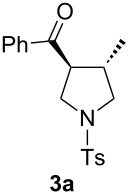	4.1:1	76
2	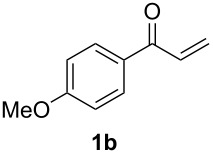	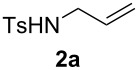	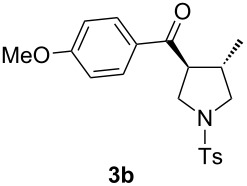	1.0:1	92
3	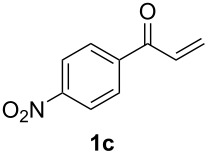	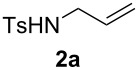	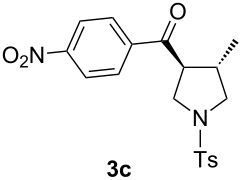	1.7:1	58
4	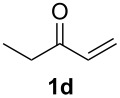	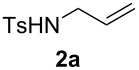	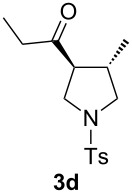	5.5:1	92
5	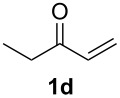	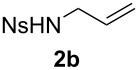	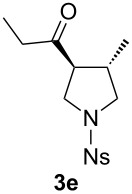	5.3:1	76
6^c^	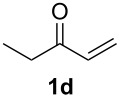	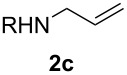	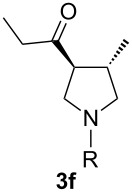	5.2:1	91
7	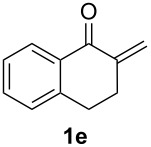	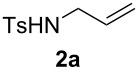	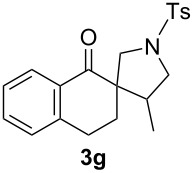	1.8:1	52

^a^Reactions were carried out in toluene (0.5 mL) at 0.25 mmol scale. The α,β-unsaturated ketone (0.25 mmol) and the substituted allyl amine (0.375 mmol) were added in one portion. ^b^Yield and selectivity were determined by ^1^H NMR spectroscopy (internal standard: trimethyl(phenyl)silane). ^c^R = 2,4,6-triisopropylbenzenesulfonyl.

**Scheme 2:**
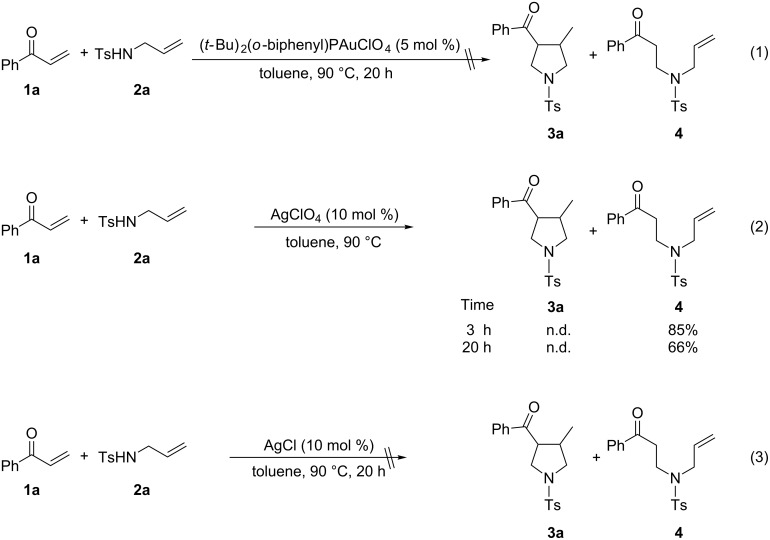
Some control experiments.

To gain insight into the mechanism of the gold(I)/silver(I)-cocatalyzed cascade reaction, we first examined the reaction of phenyl vinyl ketone (**1a**) with 1.5 equiv of *N*-tosylallylamine (**2a**) in the presence of 5 mol % of (*t*-Bu)_2_(*o*-biphenyl)PAuClO_4_ at 90 °C in toluene for 20 h, however, no desired product **3a** or α-ketone intermediate **4** was observed by ^1^H NMR analysis of the reaction mixture (Scheme 2, reaction 1). This finding revealed that the gold(I) complex is ineffective in the catalysis of the intermolecular N-Michael reaction. Upon subsequent treatment of phenyl vinyl ketone (**1a**) with *N*-tosylallylamine (**2a**) in the presence of 10 mol % of AgClO_4_ at 90 °C for 3 h, the α-ketone intermediate **4** was formed in 85% yield, however, no product **3a** was observed. Even after a longer reaction time (20 h) under the same reaction conditions, **3a** was also not detected, and α-ketone intermediate **4** was isolated in lower yield (66%) (Scheme 2, reaction 2), which may be due to the *retro*-N-Michael reaction [47]. On the other hand, product **3a** and α-ketone intermediate **4** were not observed in the presence of 10 mol % of AgCl under the same reaction conditions (Scheme 2, reaction 3), revealing that the newly formed AgCl from the reaction of (*t*-Bu)_2_(*o*-biphenyl)PAuCl and AgClO_4_ did not affect the reaction. All the results demonstrated the dual roles of the silver salt that serves firstly to abstract the coordinated Cl^−^ ligand, to give a reactive gold catalyst, and secondly to act as an efficient catalyst for the intermolecular N-Michael addition.

**Scheme 3:**
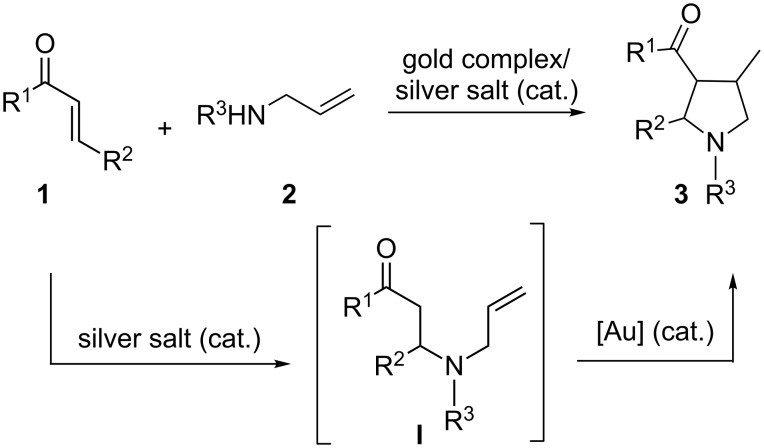
The reaction pathway.

On the basis of these observations and our previous work on gold(I)-catalyzed intramolecular hydroalkylation of unactivated alkenes with α-ketones [24], a reaction mechanism for the formation of pyrrolidine **3** from the reaction of α,β-unsaturated ketone **1** with substituted allylamine **2** is proposed (Scheme 3), which involves silver-catalyzed intermolecular N-Michael addition of substituted allylamine **2** to α,β-unsaturated ketone **1** to generate the α-ketone intermediate **I** and subsequent gold(I)-catalyzed intramolecular hydroalkylation of the α-ketone intermediate **I** to form the cyclic compound **3** (Scheme 3).

## Conclusion

In summary, we have developed a simple and efficient gold(I)/silver(I)-cocatalyzed cascade intermolecular N-Michael addition/intramolecular hydroalkylation reaction. The present protocol with a dual-metal catalytic system provides a highly efficient method for the synthesis of a variety of pyrrolidine compounds in moderate to excellent product yields and with moderate to good diastereoselectivities from α,β-unsaturated ketones and substituted allylamines. Further studies to expand the substrate scope are currently in progress.

## Supporting Information

File 1 Experimental section and spectra of compounds.
