# Supplementary material for: Efficient gold(I)/silver(I)-cocatalyzed cascade intermolecular N-Michael addition/intramolecular hydroalkylation of unactivated alkenes with α-ketones
Source: Beilstein J Org Chem. 2011 Aug 11;7:1100–7. doi: 10.3762/bjoc.7.126 (PMC3170194; doi:10.3762/bjoc.7.126)
Supplement: File 1 — Experimental section and spectra of compounds. [file Beilstein_J_Org_Chem-07-1100-s001.pdf]

Supporting Information

for

Efficient gold(I)/silver(I)-cocatalyzed cascade intermolecular N-Michael addition/intramolecular hydroalkylation of unactivated alkenes with α-ketones

Ya-Ping Xiao[1], Xin-Yuan, Liu[2], Chi-Ming Che[1,2]*

Address: [1]Shanghai-Hong Kong Joint Laboratory in Chemical Synthesis, Shanghai Institute of Organic Chemistry, The Chinese Academy of Sciences, 345 Lingling Road, Shanghai 200032 , P. R. China and [2]Department of Chemistry, State Key Laboratory of Synthetic Chemistry, and Open Laboratory of Chemical Biology of the Institute of Molecular Technology for Drug Discovery and Synthesis, The University of Hong Kong, Pokfulam Road, Hong Kong, P. R. China

Email: Chi-Ming Che* - cmche@hku.hk; Ya-Ping Xiao - xiaoyaping82@hotmail.com; Xin-Yuan Liu - liuxy@hku.hk

*Corresponding author

Experimental section and spectra of compounds

Table of contents

Experimental section S2

References S9

Spectra of compounds S10–S17

Experimental section

General methods. Reagents were obtained commercially and used without further purification unless indicated otherwise. All anhydrous solvents used in the reactions were dried and freshly distilled. All manipulations with air-sensitive reagents were carried out under a dry argon atmosphere. The catalysts $Au(PPh_3)Cl$ [1], $(Cy)_2$(2',4',6'-triisopropyl-*o*-biphenyl)PAuCl [1,2], (*t*-Bu)$_2$(*o*-diphenyl)PAuCl [1,2] and IPrAuCl [3] were prepared following literature procedures. 2-Methylene-3,4-dihydronaphthalen-1(*2H*)-one was prepared according to the literature procedure [4]. α,β-Unsaturated ketones were prepared following the literature procedure [5]. Substituted allylic amines were prepared following the literature procedure [6]. NMR spectra were recorded on Bruker AM300/400 spectrometers at 300/400 MHz for ^{1}H NMR and 75/100 MHz for ^{13}C NMR in $CDCl_3$ with TMS as an internal standard. The chemical shifts are expressed in ppm and coupling constants are given in Hz. Data for ^{1}H NMR are recorded as follows: Chemical shift (ppm), multiplicity (s, singlet; d, doublet; t, triplet; q, quartet; m, multiplet), coupling constant (Hz), integration. Data for ^{13}C NMR are reported in terms of chemical shift (δ, ppm). Mass spectra were obtained on a HP5989A spectrometer (EI), an IonSpec 4.7 Tesla FTMS spectrometer (MALDI), or a Bruker

Daltonics FTMS-7 spectrometer (ESI). IR spectra were recorded as KBr discs, on a Bio-Rad FTS-185 spectrometer; frequencies are given in reciprocal centimeters (cm^{-1}) and only selected absorbance is reported.

General procedure for gold/silver-cocatalyzed one-pot tandem intermolecular N-Michael addition/intramolecular hydroalkylation

A mixture of (*t*-Bu)$_2$(*o*-diphenyl)PAuCl (6.7 mg, 0.0125 mmol), $AgClO_4$ (7.8 mg, 0.0375 mmol) (*Warning! The perchlorate salt is potentially explosive and should be handled with great caution.*), α,β-unsaturated ketone (0.25 mmol) and substituted allylic amine (0.375 mmol, 1.5 equiv) in toluene (0.5 mL) was stirred at 90 °C under Ar atmosphere for 20 h. Upon completion, the solvent was removed under reduced pressure, and the residue was purified by silica gel column chromatography (eluent: EtOAc/petroleum ether = 1:12-1:6) to give the desired products.

(*trans*-4-Methyl-1-tosylpyrrolidin-3-yl)(phenyl)methanone (3a)

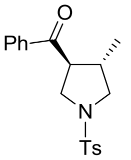

3a

trans/*cis*: 4.1:1. Major diastereomer could be separated on a silica gel column, and the relative configuration of **3a** was determined with reference to 1-(*trans*-4-methyl-1-tosylpyrrolidin-3-yl)ethanone [7].

White solid. ^{1}H NMR(300 MHz, $CDCl_3$): δ 7.86 (d, J = 7.3 Hz, 2H), 7.72 (d, J = 8.2 Hz, 2H), 7.60 (t, J = 7.3 Hz, 1H), 7.48 (t, J = 7.6 Hz, 2H), 7.34 (d, J = 7.3 Hz, 2H), 3.79 (t, J = 8.2 Hz, 1H), 3.55 (q, J = 8.0 Hz, 1H), 3.47 (dd, J = 7.2, 9.0 Hz, 1H), 3.29 (t, J = 9.0 Hz, 1H), 3.05 (t, J = 7.6 Hz, 1H), 2.56-2.46 (m, 1H), 2.44 (s, 3H), 1.04 (d, J = 6.7 Hz, 3H); ^{13}C NMR (75 MHz, $CDCl_3$): δ 198.1, 143,6, 136.2, 133.7, 133.4, 129.7,

128.8, 128.3, 127.6, 54.2, 50.8, 36.5, 21.6, 17.4. IR (FILM): ν_{max} 3286, 2956, 2924, 1712, 1679, 1597, 1448, 1341, 1223, 1161, 1093, 1041, 815 cm^{-1}. MS (ESI) *m*/*z*: 366 ($M+Na^+$), 344 ($M+H^+$). HRMS (ESI): calcd. for $C_{19}H_{22}NO_3S^+$ ($M+H^+$): 344.13149, found: 344.13189.

(4-Methoxyphenyl)(*trans*-4-methyl-1-tosylpyrrolidin-3-yl)methanone (3b)

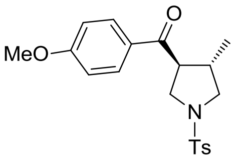

3b

trans/cis: 1.0: 1. One of the diastereomers could be separated on a silica gel column, and the relative configuration of **3b** was determined with reference to **3a**.

White solid. ^{1}H NMR (300 MHz, $CDCl_3$): δ 7.84 (d, *J* = 8.4Hz, 2H), 7.71 (d, *J* = 7.5Hz, 2H), 7.33 (d, *J* = 7.5Hz, 2H), 6.93 (d, *J* = 8.4Hz, 2H), 3.87 (s, 3H), 3.75 (t, *J* = 8.7 Hz, 1H), 3.54-3.43 (m, 2H), 3.27 (t, *J* = 9.0Hz, 1H), 3.04 (t, *J* = 8.7Hz, 1H), 2.54-2.44 (m, 1H), 2.44(s, 3H), 1.01 (d, *J* = 6.9 Hz, 3H); ^{13}C NMR (75 MHz, $CDCl_3$): δ 196.4, 163.9, 143.6, 133.4, 130.7, 129.7, 129.2, 127.5, 113.9, 55.5, 54.2, 51.6, 51.0, 36.6, 21.5, 17.3; MS (ESI) *m*/*z*: 396 ($M+Na^+$); HRMS (ESI): calcd. for $C_{20}H_{23}NNaO_4S^+$ ($M+Na^+$): 396.12400, found: 396.12381.

(*cis*-4-Methyl-1-tosylpyrrolidin-3-yl)(4-nitrophenyl)methanone (3c)

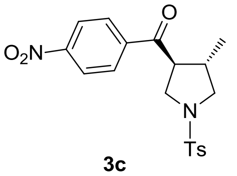

3c

trans/cis: 1.7: 1. One of the diastereomers could be separated on a silica gel column, and the relative configuration of **3c** was determined with reference to **3a**.

Pale yellow solid. ^{1}H NMR (300 MHz, $CDCl_3$): δ 8.32 (d, *J* = 8.4Hz, 2H), 8.04 (d, *J* = 8.4Hz, 2H), 7.77 (d, *J* = 7.8 Hz, 2H), 7.37 (d, *J* = 7.8 Hz, 2H), 4.07-3.99 (m, 1H),

3.75-3.58 (m, 3H), 3.08 (dd, J = 9.6, 3.6 Hz, 1H), 2.76-2.72 (m, 1H), 2.47 (s, 3H), 0.57 (d, J = 7.2 Hz, 3H); ^{13}C NMR (100 MHz, $CDCl_3$): δ 196.9, 150.9, 144.1, 141.1, 133.8, 128.0, 124.5, 55.4, 49.9, 47.9, 36.6, 21.9, 14.9; IR (FILM): ν_{max} 3108, 2967, 2925, 1688, 1602, 1526, 1493, 1407, 1346, 1221, 1164, 1093, 1032, 985 cm^{-1}; MS (ESI) m/z: 411(M+Na$^+$); HRMS (ESI): calcd. for $C_{19}H_{20}N_2NaO_5S^+$ (M+Na$^+$): 411.10102, found: 411.09913.

1-(*trans*-4-Methyl-1-tosylpyrrolidin-3-yl)propan-1-one (3d)

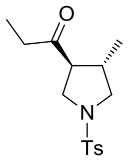

3d

trans/*cis*: 5.5:1. Major diastereomer could be separated on a silica gel column, and the relative configuration of **3d** was determined with reference to **3a**.

White solid. ^{1}H NMR (300 MHz, $CDCl_3$): δ 7.71 (d, J = 8.1Hz, 2H), 7.34 (d, J = 8.1Hz, 2H), 3.60 (dd, J = 9.6, 8.4Hz, 1H), 3.41 (dd, J = 9.6, 7.5Hz, 1H), 3.24 (dd, J = 9.9, 8.4Hz, 1H), 2.90 (dd, J = 9.6, 8.1Hz, 1H), 2.69 (dd, J = 16.5, 8.1Hz, 1H), 2.45 (s, 3H), 2.45-2.23 (m, 3H), 1.04-0.99 (m, 6H); ^{13}C NMR (75 MHz, $CDCl_3$): δ 209.1, 143.7, 133.3, 129.7, 127.6, 56.7, 54.3, 49.9, 36.3, 36.1, 21.5, 17.5, 7.5; IR (FILM): ν_{max} 2972, 2937, 2877, 1713, 1598, 1459, 1379, 1343, 1162, 1093 cm^{-1}; MS (ESI) m/z: 318 (M+Na$^+$), 296 (M+H$^+$); HRMS (ESI): calcd. for $C_{15}H_{21}NNaO_3S^+$ (M+Na$^+$): 318.11344, found: 318.11249.

1-(*trans*-4-Methyl-1-(4-nitrophenylsulfonyl)pyrrolidin-3-yl)propan-1-one (3e)

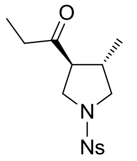

3e

trans/*cis*: 5.3:1. Major diastereomer could be separated on a silica gel column, and the relative configuration of **3e** was determined with reference to **3a**.

Pale yellow solid. ^{1}H NMR (300 MHz, $CDCl_3$): δ 8.38 (d, *J* = 8.7Hz, 2H), 8.00 (d, *J* = 8.7Hz, 2H), 3.60 (dd, *J* = 9.9, 1.8Hz, 1H), 3.38-3.31 (m, 2H), 2.99 (dd, *J* = 9.6, 7.2Hz, 1H), 2.77 (dd, *J* = 15.0, 7.5Hz, 1H), 2.46-2.39 (m, 2H), 2.38-2.28 (m, 1H), 1.05 (d, *J* = 6.6Hz, 3H), 0.99 (t, *J* = 7.2Hz, 3H); ^{13}C NMR (75 MHz, $CDCl_3$): δ 209.2, 150.4, 142.9, 128.9, 124.7, 56.6, 54.5, 49.8, 36.9, 36.4, 17.9, 7.8; MS (EI) *m*/*z*: 326 (M^+, 1), 140 (100), 122 (13), 113 (48), 108 (12), 85 (15), 84 (60), 82(41); HRMS (EI): calcd. for $C_{12}H_{13}N_2O_5S^+$ (M^+): 297.0545, found: 297.0542.

1-(*trans*-4-Methyl-1-(2,4,6-triisopropylphenylsulfonyl)pyrrolidin-3-yl)propan-1-one (3f)

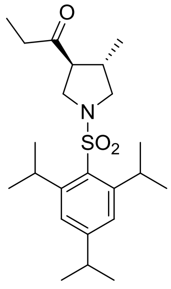

3f

trans/*cis*: 5.2:1. Major diastereomer could be separated on a silica gel column, and the relative configuration of **3f** was determined with reference to **3a**.

Pale yellow solid. ^{1}H NMR (400 MHz, $CDCl_3$): δ 7.15 (s, 2H), 4.21-4.15 (m, 2H), 3.63 (dd, *J* = 7.2, 6.3Hz, 1H), 3.41 (dd, *J* = 6.9, 5.4Hz, 1H), 3.31 (dd, *J* = 7.5, 6.3Hz, 1H), 2.97-2.78 (m, 3H), 2.51-2.44 (m, 3H), 1.27-1.22 (m, 18H), 1.09 (d, *J* = 5.1Hz,

3H), 1.04 (t, *J* = 5.4Hz, 3H)； ^{13}C NMR(100 MHz, $CDCl_3$): δ 209.6, 153.1, 151.2, 131.0, 123.8, 57.0, 53.2, 48.6, 36.7, 36.1, 34.1, 29.3, 24.8, 23.5, 17.3, 7.5; MS (EI) *m*/*z*: 407 (M^+, 1), 306 (14), 268 (18), 267 (100), 251 (32), 249 (9), 218 (24), 203 (14); HRMS (EI): calcd. for $C_{23}H_{37}NO_3S^+$ (M^+): 407.2494, found: 407.2487.

4'-Methyl-1'-tosyl-3,4-dihydro-1*H*-spiro[naphthalene-2,3'-pyrrolidin]-1-one (3g)

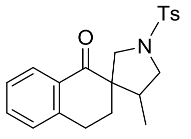

3g

trans/*cis*: 1.8: 1. One of diastereomers could be separated on a silica gel column. White solid. ^{1}H NMR (300 MHz, $CDCl_3$) δ 7.86 (d, *J* = 7.2Hz, 1H), 7.72 (d, *J* = 8.1Hz, 2H), 7.49-7.44 (m, 1H), 7.35-7.26 (m, 3H), 7.21 (d, *J* = 7.8Hz, 2H), 3.83 (d, *J* = 9.9Hz, 1H), 3.67 (dd, *J* = 9.3, 7.2Hz, 1H), 3.27 (d, *J* = 10.5Hz, 1H), 3.13-3.02 (m, 2H), 2.94-2.85 (m, 1H), 2.46 (s, 3H), 2.39-2.32 (m, 1H), 2.25-2.17 (m, 1H), 2.08-1.99 (m, 1H), 0.71 (d, *J* = 6.9Hz, 3H); ^{13}C NMR (75 MHz, $CDCl_3$): δ 197.7, 143.4, 142.6, 133.6, 133.3, 132.0, 129.6, 128.7, 127.62, 127.57, 126.8, 55.6, 54.8, 54.2, 39.9, 33.2, 25.8, 21.6, 14.1; MS(ESI) *m*/*z*:: 392 ($M+Na^+$), 370 ($M+H^+$); HRMS (ESI): calcd. for $C_{21}H_{23}NNaO_3S^+$ ($M+Na^+$): 392.12909, found: 392.12958.

General procedure for control experiment

A mixture of $AgClO_4$ (20.7 mg, 0.1 mmol) (*Warning! The perchlorate salt is potentially explosive and should be handled with great caution.*), α,β-unsaturated ketone **1a** (1.0 mmol) and substituted allylic amine **2a** (1.5 mmol, 1.5 equiv) in toluene (2 mL) was stirred at 90 °C under Ar atmosphere for 3 h. Then, the solvent was removed under reduced pressure, and the residue was purified by silica gel column chromatography (eluent: EtOAc/petroleum ether = 1:10) to give the desired product **4** in 85% yield (299 mg, 0.85 mmol).

***N*-Allyl-4-methyl-N-(3-oxo-3-phenylpropyl)benzenesulfonamide (4)**

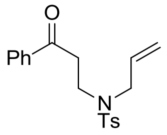

4

Colorless oil. ^{1}H NMR (300 MHz, $CDCl_3$): δ 7.93 (d, *J* = 8.0 Hz, 2H), 7.70 (d, *J* = 8.3 Hz, 2H), 7.58 (t, *J* = 7.0 Hz, 1H), 7.46 (t, *J* = 7.2 Hz, 2H), 7.28 (t, *J* = 8.6 Hz, 2H), 5.75-5.62 (m, 1H), 5.22-5.13 (m, 2H), 3.84 (d, *J* = 6.6 Hz, 2H), 3.48 (t, *J* = 6.5 Hz, 2H), 3.36 (t, *J* = 7.2 Hz, 2H), 2.44 (s, 3H); ^{13}C NMR (75 MHz, $CDCl_3$): δ 198.3, 143.4, 136.3, 136.2, 133.3, 133.0, 129.7, 128.6, 127.9, 127.1, 119.3, 52.1, 43.1, 38.9, 21.5. IR(FILM): v_{max} 3064, 2922, 1682, 1644, 1598, 1581, 1494, 1449, 1417, 1382, 1342, 1306, 1287, 1211, 1157, 1092, 1018, 986, 931, 876 cm^{-1}. MS(ESI) *m*/*z*: 366 ($M+Na^+$), 344 ($M+H^+$). HRMS(ESI): calcd. for $C_{19}H_{21}NO_3SNa^+$($M+Na^+$): 366.1134, found: 366.1141. Anal. calcd. for $C_{19}H_{21}O_3NS$: C, 66.45; H, 6.16; N, 4.08, found: C, 66.45; H, 6.10; N, 4.05.

References

1. Al-Sa'Ady, A. K.; McAuliffe, C. A.; Parish, R. V.; Sandeank, J. A. *Inorg. Synth.* **1985,** 191–194.
2. Nieto-Oberhuber, C.; López, S.; Echavarren, A. M. *J. Am. Chem. Soc.* **2005,** *127,* 6178–6179. doi:10.1021/ja042257t
3. de Frémont, P.; Scott, N. M.; Stevens, E. D.; Nolan, S. P. *Organometallics* **2005,** *24*, 2411–2418. doi:10.1021/om050111c
4. Gras, J. L. *Org. Synth. Coll.***1990,** *Vol. 7,* 332.
5. Barluenga, J.; Fanlo, H.; López, S.; Flórez, J. *Angew. Chem., Int. Ed.* **2007,** *46,* 4136–4140. doi:10.1002/anie.200605167
6. Taillier, C.; Hameury, T.; Bellosta, V.; Cossy, J. *Tetrahedron* **2007,** *63,* 4472–4490. doi:10.1016/j.tet.2007.03.066
7. Xiao, Y.-P.; Liu, X.-Y.; Che, C.-M. *Angew. Chem., Int. Ed.* **2011,** *50,* 4937–4941. doi:10.1002/anie.201100044

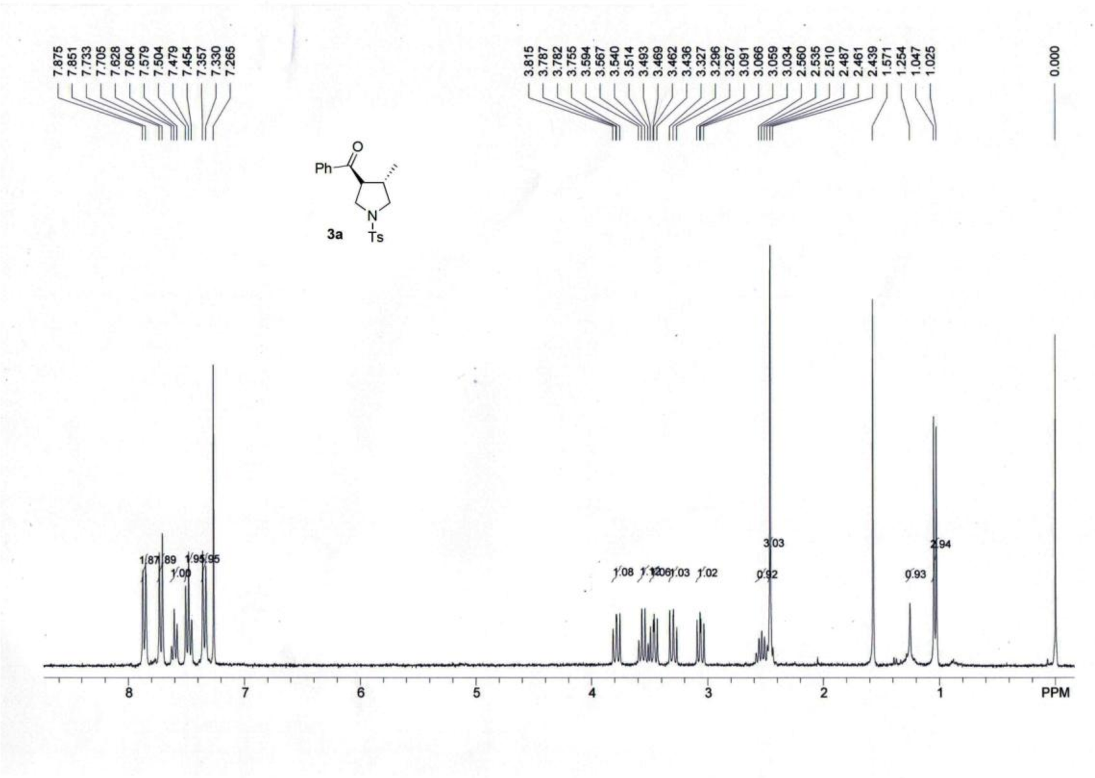

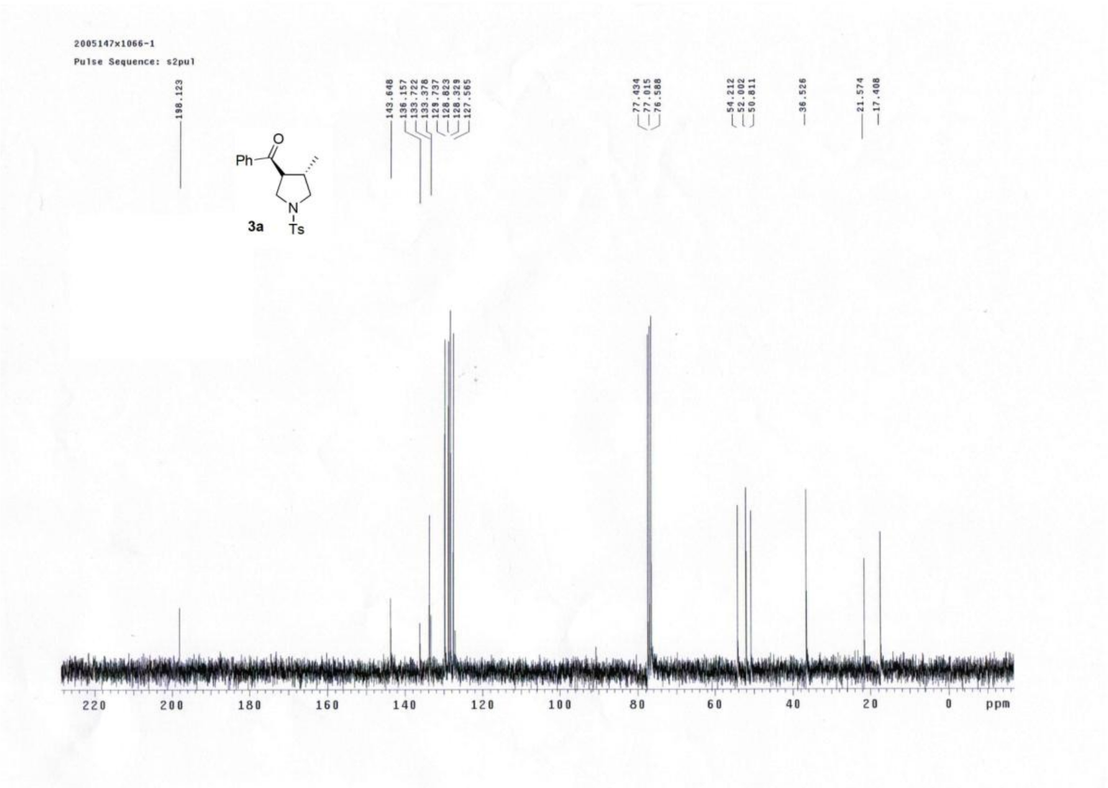

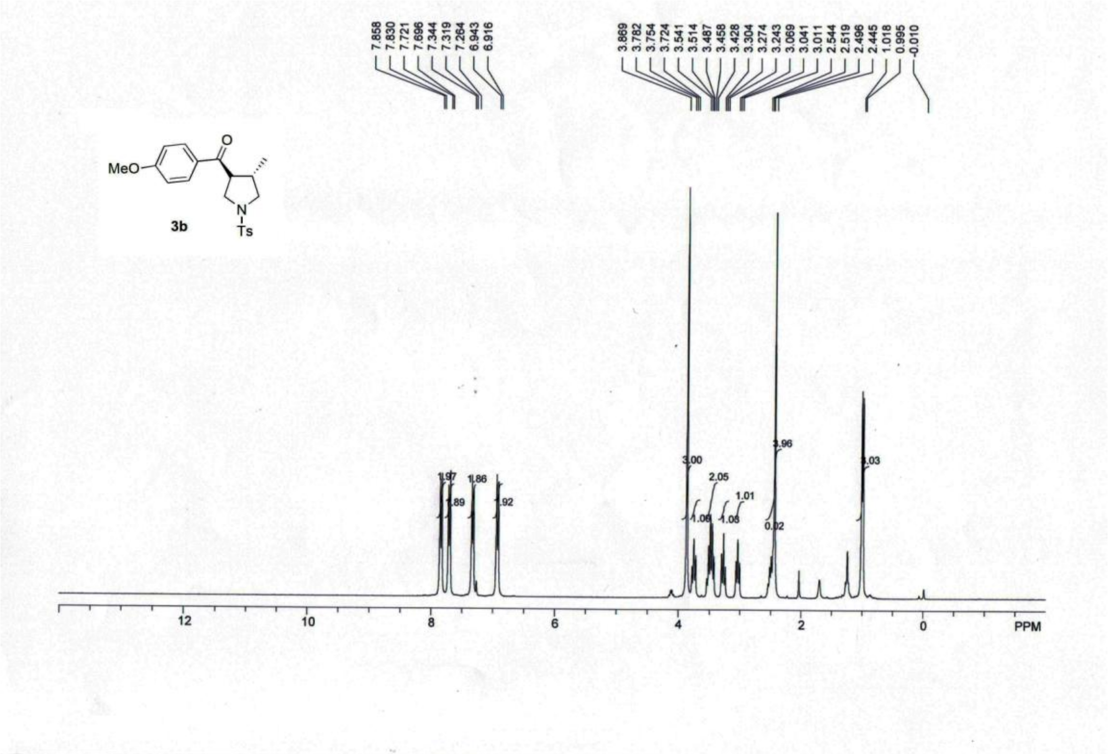
MeO
3b
Ts
PPM

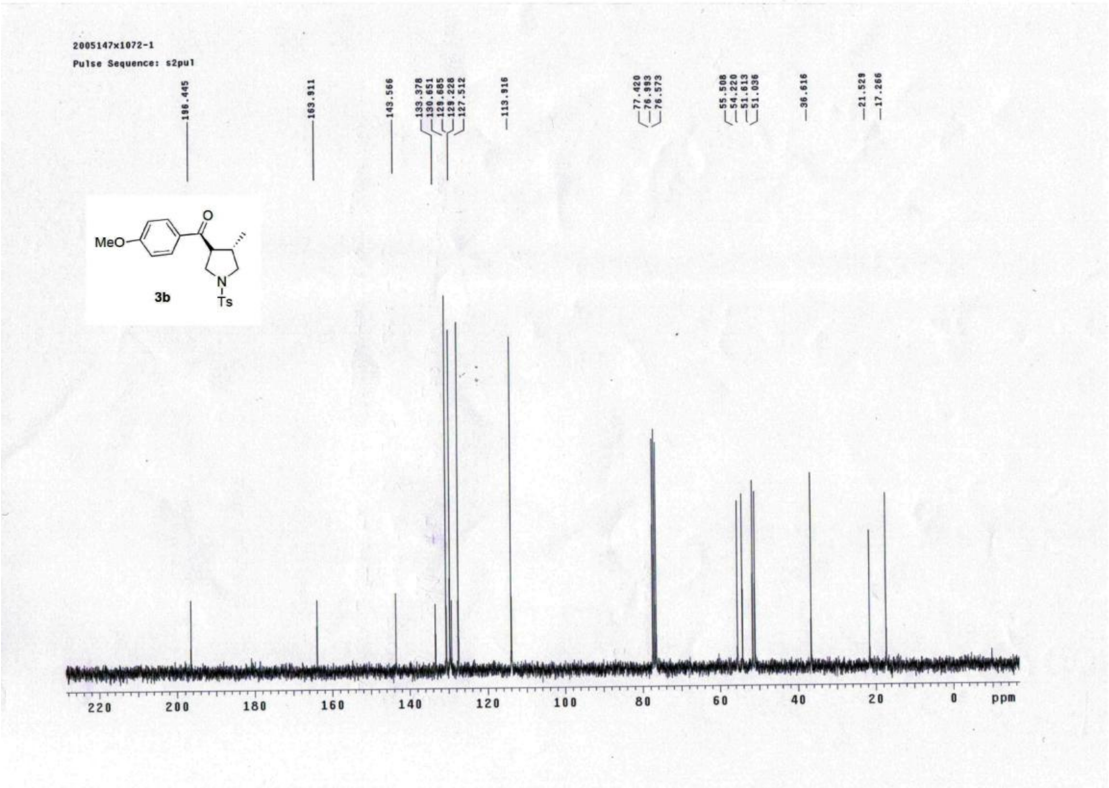
2005147x1072-1
Pulse Sequence: s2pul
MeO
3b
Ts
ppm

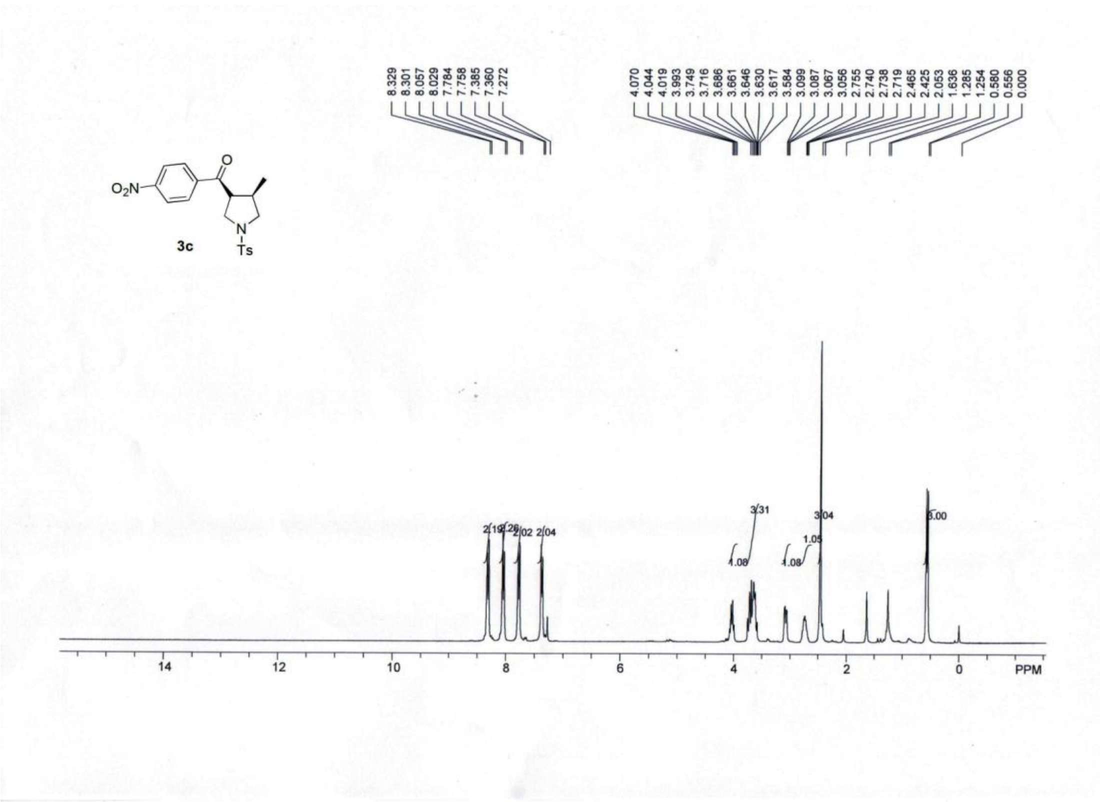

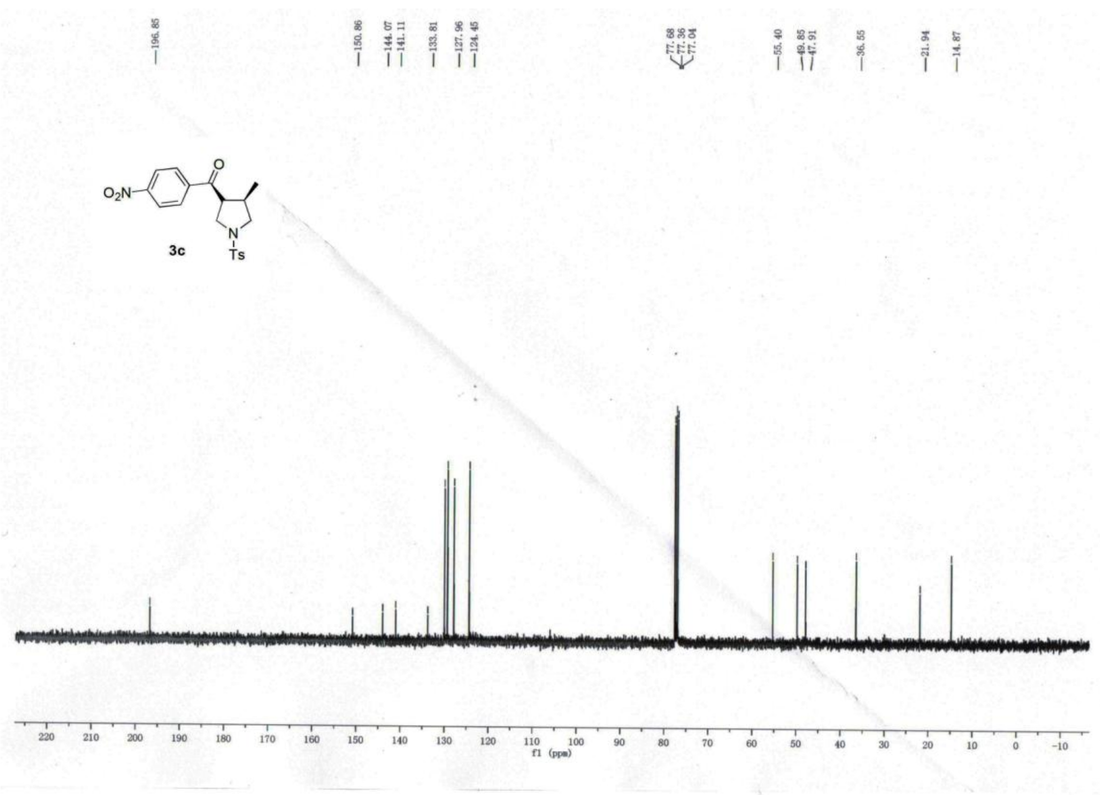

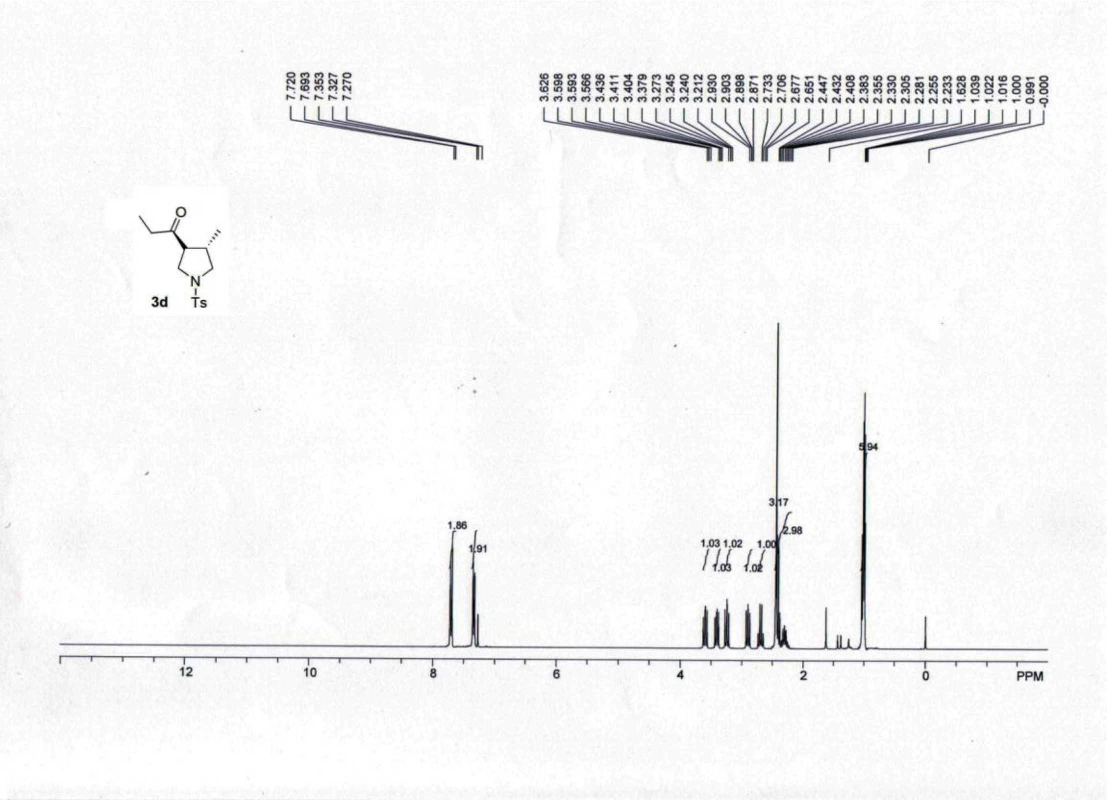

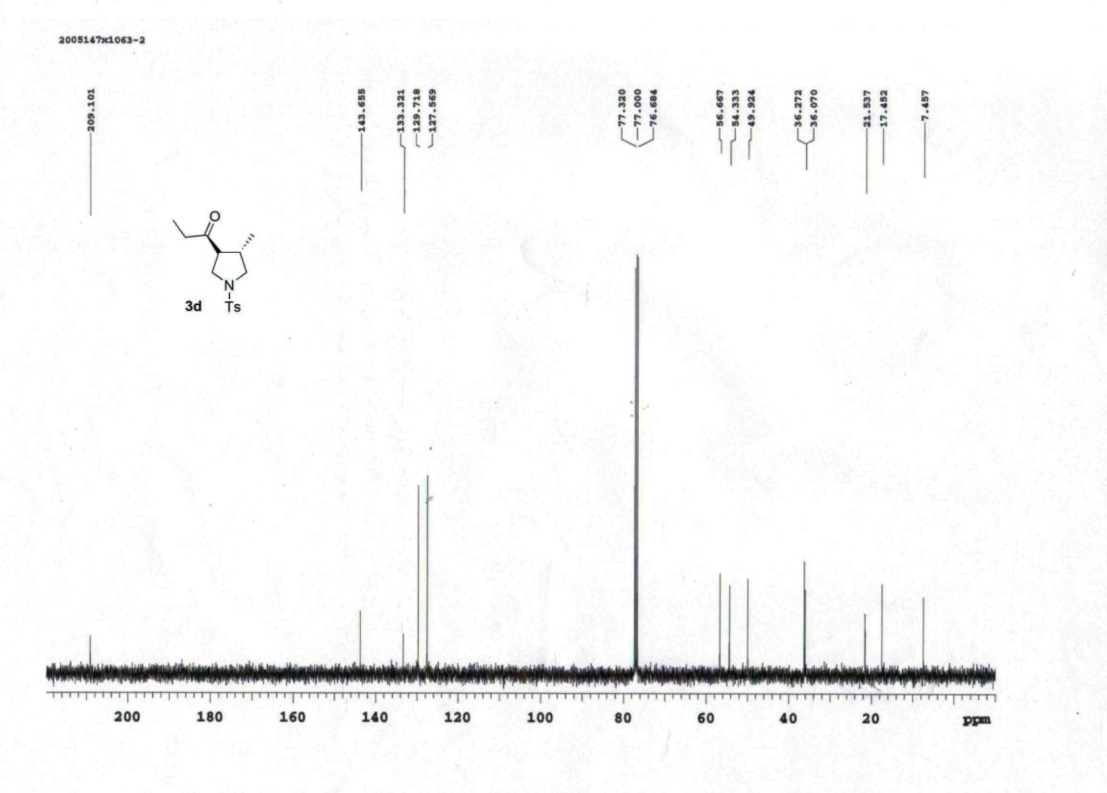

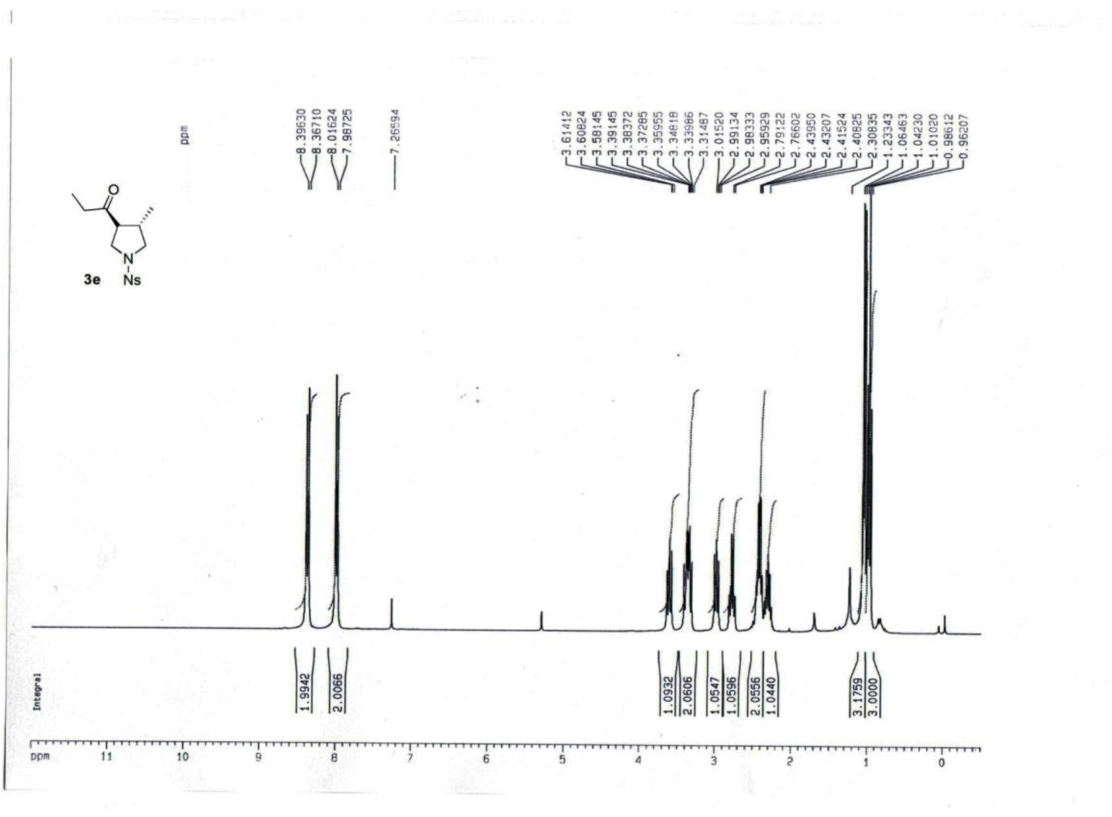
3e
Ns
ppm
8.39630
8.36710
8.01624
7.98725
7.26594
3.61412
3.60824
3.58145
3.39145
3.38372
3.37285
3.35955
3.34818
3.33986
3.31487
3.01520
2.99134
2.98333
2.95929
2.79122
2.76602
2.43950
2.43207
2.41524
2.40825
2.30835
1.23343
1.06463
1.04230
1.01020
0.98612
0.96207
Integral
1.9942
2.0066
1.0932
2.0606
1.0547
1.0596
2.0556
1.0440
3.1759
3.0000

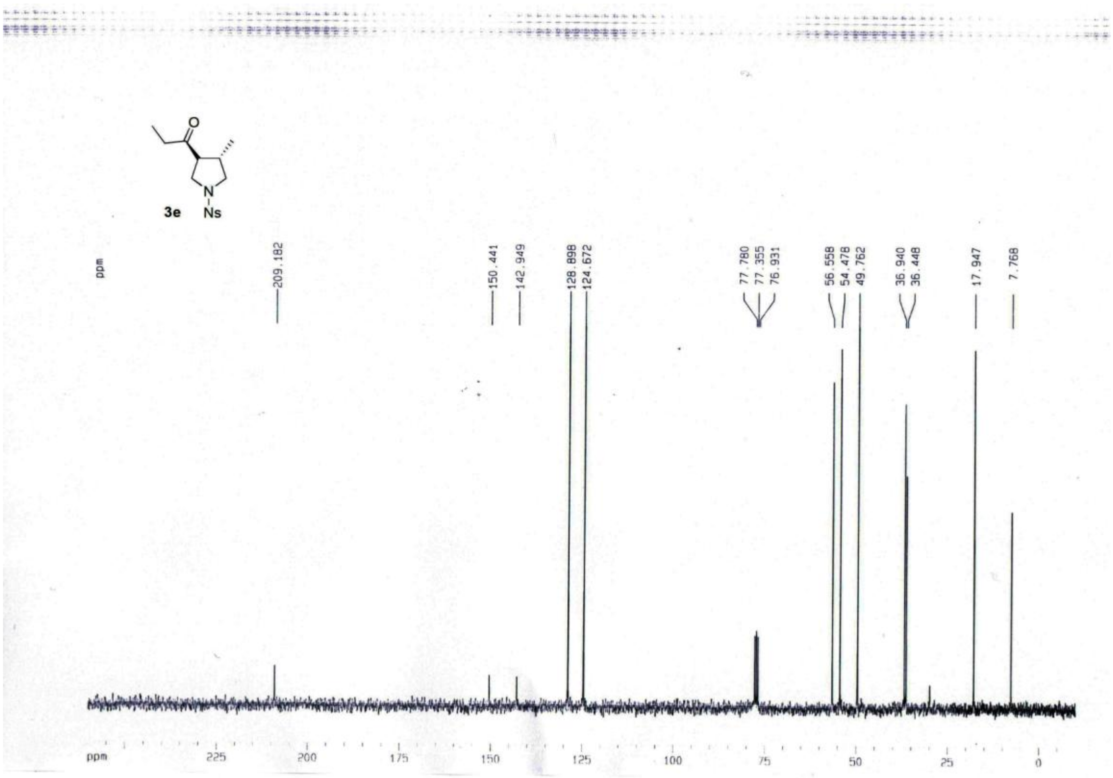
3e
Ns
ppm
209.182
150.441
142.949
128.898
124.672
77.780
77.355
76.931
56.558
54.478
49.762
36.940
36.448
17.947
7.768

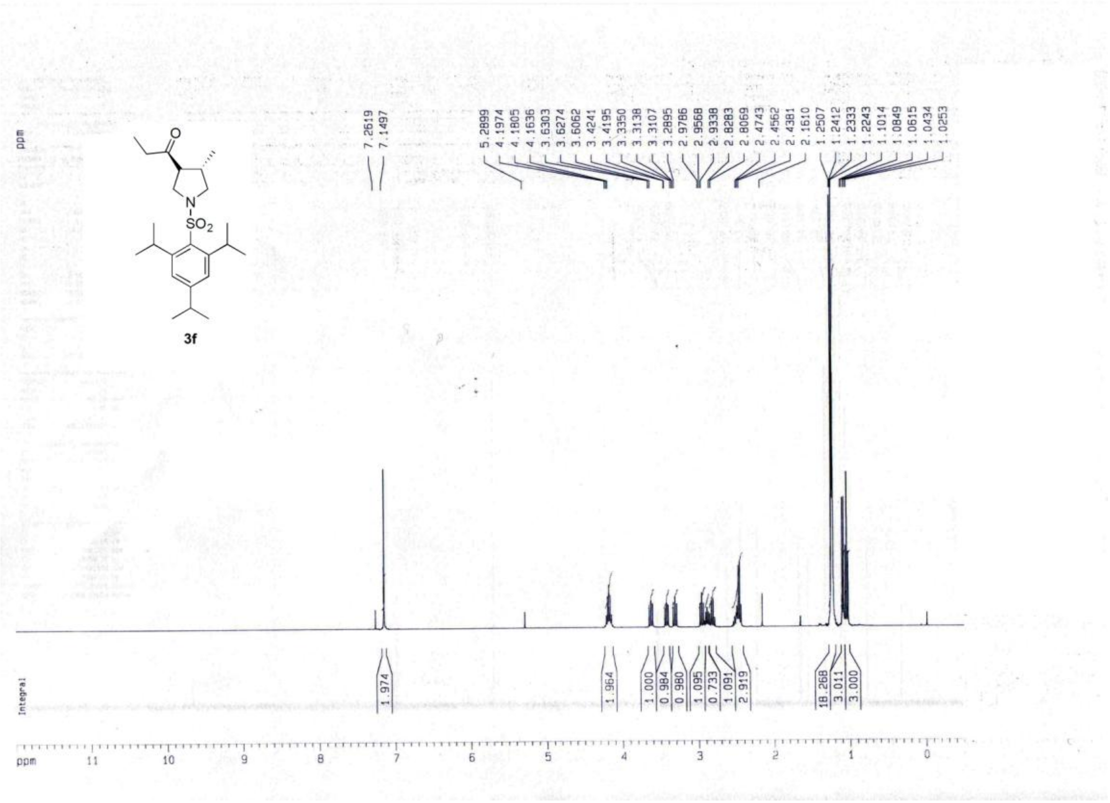
ppm
3f
7.2619
7.1497
5.2899
4.1974
4.1805
4.1636
3.6303
3.6274
3.6062
3.4241
3.4195
3.3350
3.3138
3.3107
3.2895
2.9786
2.9568
2.9338
2.8283
2.8069
2.4743
2.4562
2.4381
2.1610
1.2507
1.2412
1.2333
1.2243
1.1014
1.0849
1.0615
1.0434
1.0253
Integral
1.974
1.964
1.000
0.984
0.980
1.095
0.733
1.091
2.919
18.268
3.011
3.000
ppm
11
10
9
8
7
6
5
4
3
2
1
0

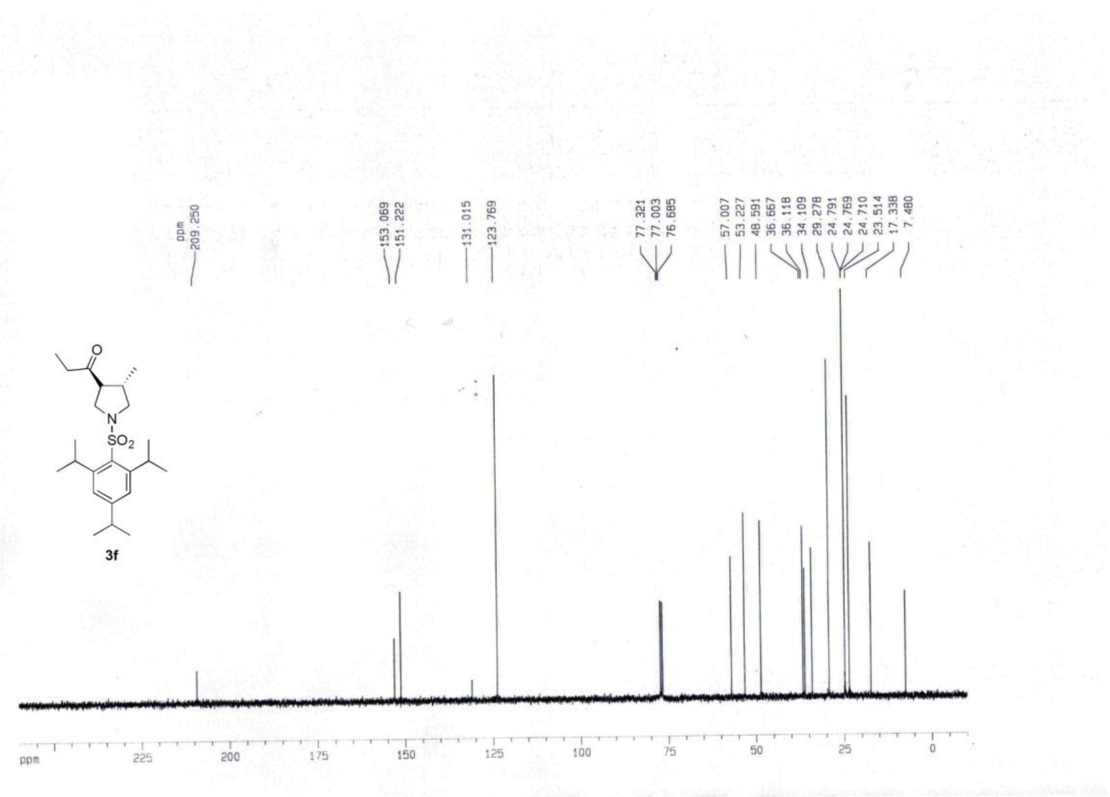
ppm
209.250
153.069
151.222
131.015
123.769
77.321
77.003
76.685
57.007
53.227
48.591
36.667
36.118
34.109
29.278
24.791
24.769
24.710
23.514
17.338
7.480
3f
ppm
225
200
175
150
125
100
75
50
25
0

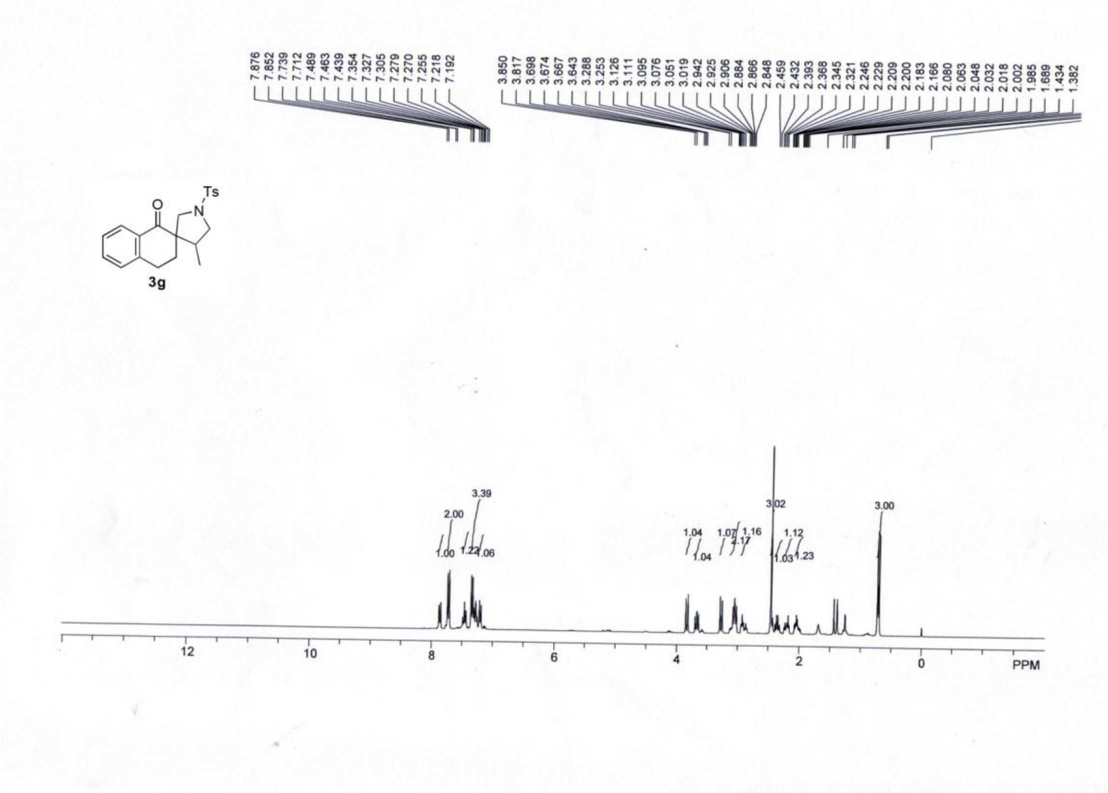
3g
PPM

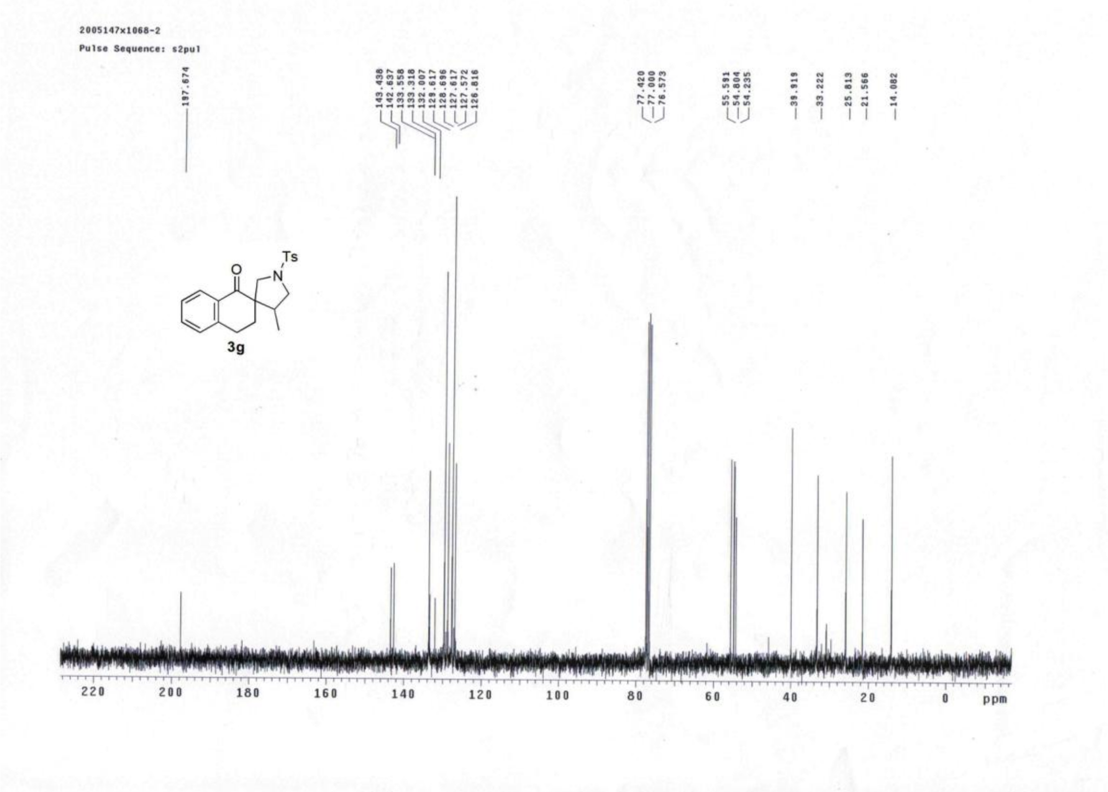
2005147x1068-2
Pulse Sequence: s2pul
3g
ppm

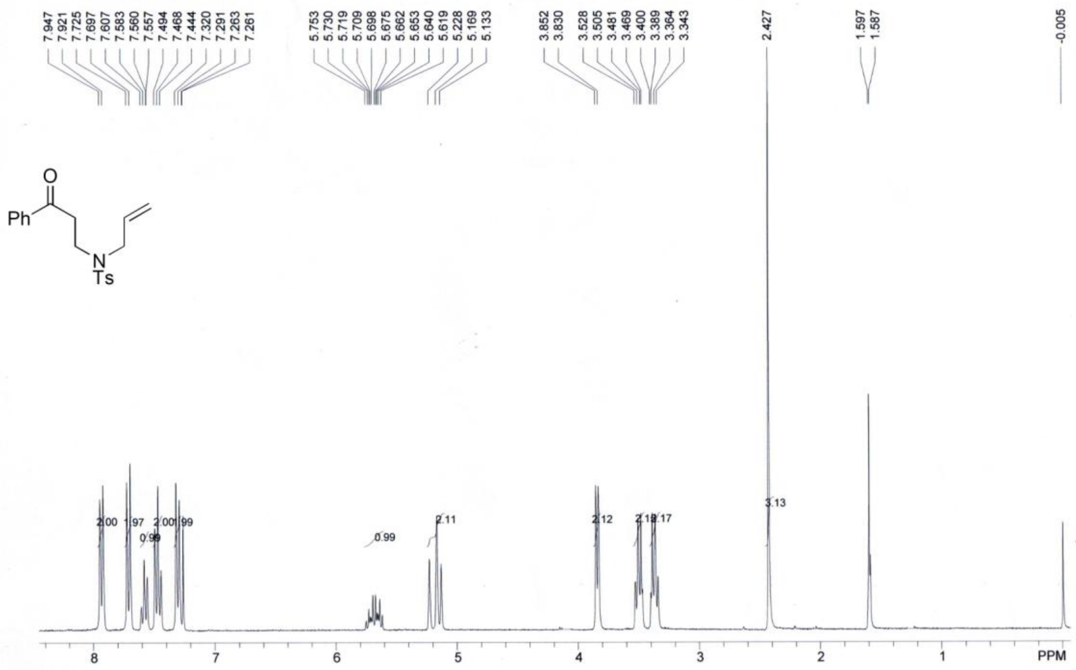

Ph
N
Ts
PPM

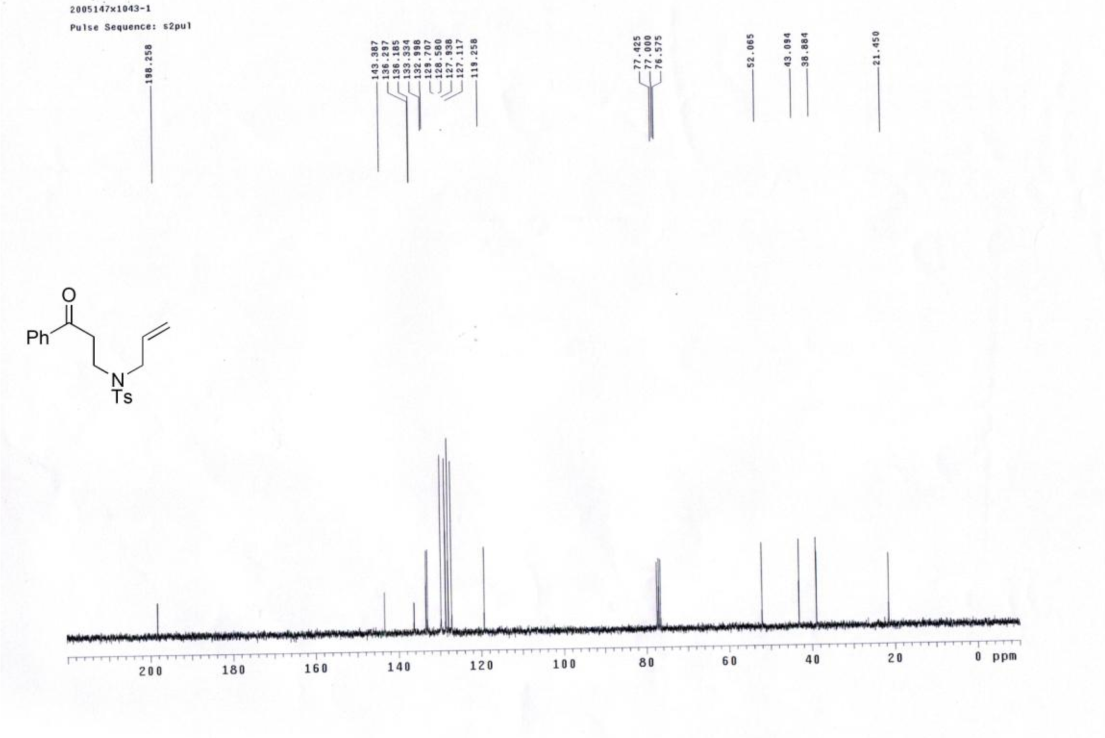

2005147x1043-1
Pulse Sequence: s2pul
Ph
N
Ts
ppm
